# Large-scale drivers of malaria and priority areas for prevention and control in the Brazilian Amazon region using a novel multi-pathogen geospatial model

**DOI:** 10.1186/1475-2875-13-443

**Published:** 2014-11-20

**Authors:** Denis Valle, Joanna M Tucker Lima

**Affiliations:** School of Forest Resources and Conservation, University of Florida, 408 McCarty Hall C, PO Box 110339, Gainesville, FL USA

**Keywords:** Malaria, Brazilian amazon, Bayesian model, Geospatial model, Hotspot, *Plasmodium falciparum*, Malaria risk maps, Forest cover, Gold mining, Migration

## Abstract

**Background:**

Most of the malaria burden in the Americas is concentrated in the Brazilian Amazon but a detailed spatial characterization of malaria risk has yet to be undertaken.

**Methods:**

Utilizing 2004-2008 malaria incidence data collected from six Brazilian Amazon states, large-scale spatial patterns of malaria risk were characterized with a novel Bayesian multi-pathogen geospatial model. Data included 2.4 million malaria cases spread across 3.6 million sq km. Remotely sensed variables (deforestation rate, forest cover, rainfall, dry season length, and proximity to large water bodies), socio-economic variables (rural population size, income, and literacy rate, mortality rate for children age under five, and migration patterns), and GIS variables (proximity to roads, hydro-electric dams and gold mining operations) were incorporated as covariates.

**Results:**

Borrowing information across pathogens allowed for better spatial predictions of malaria caused by Plasmodium falciparum, as evidenced by a ten-fold cross-validation. Malaria incidence for both Plasmodium vivax and P. falciparum tended to be higher in areas with greater forest cover. Proximity to gold mining operations was another important risk factor, corroborated by a positive association between migration rates and malaria incidence. Finally, areas with a longer dry season and areas with higher average rural income tended to have higher malaria risk. Risk maps reveal striking spatial heterogeneity in malaria risk across the region, yet these mean disease risk surface maps can be misleading if uncertainty is ignored. By combining mean spatial predictions with their associated uncertainty, several sites were consistently classified as hotspots, suggesting their importance as priority areas for malaria prevention and control.

**Conclusion:**

This article provides several contributions. From a methodological perspective, the benefits of jointly modelling multiple pathogens for spatial predictions were illustrated. In addition, maps of mean disease risk were contrasted with that of statistically significant disease clusters, highlighting the critical importance of uncertainty in determining disease hotspots. From an epidemiological perspective, forest cover and proximity to gold mining operations were important large-scale drivers of disease risk in the region. Finally, the hotspot in Western Acre was identified as the area that should receive highest priority from the Brazilian national malaria prevention and control programme.

**Electronic supplementary material:**

The online version of this article (doi:10.1186/1475-2875-13-443) contains supplementary material, which is available to authorized users.

## Background

The Brazilian Amazon region plays a critical role in the Americas, both in terms of the number of malaria cases and fatalities. Approximately 40% of deaths due to malaria in the Americas occurred in Brazil alone [1] and nearly all malaria cases in Brazil originate from within the Amazon region [1, 2]. Despite the existence of extensive data regularly collected by the Brazilian malaria surveillance system, analyses of large-scale patterns of malaria incidence and its drivers in the Brazilian Amazon are rare. For instance, Olson *et al.* [3] found that the relationship between precipitation and malaria incidence tends to be negative in areas with extensive wetlands, whereas no discernible pattern was evident in counties with few or no wetlands. Hahn *et al.* [4] revealed that roads, forest fires and selective logging are important risk factors for malaria, but they found no association between deforestation rate and malaria. On the other hand, Achcar *et al*. [5] found a positive relationship between deforestation rate and malaria incidence as well as a negative relationship between malaria incidence and human development index and population density. Finally, Valle and Clark [6] found that higher malaria incidence is frequently observed in areas with greater forest cover. Determining the key drivers of malaria risk in this region is particularly important given current and future large-scale environmental transformations due to ongoing expansion of the road network and the construction of multiple hydro-electric dams [7, 8].

Currently, the spatial characterization of malaria risk in the Brazilian Amazon is based on the calculation of the incidence per year per thousand inhabitants (i.e., annual parasite index - API) for each county, which is categorized into low, medium and high API groups and displayed in choropleth maps [2, 9, 10]. These results are used by the Brazilian Health Ministry to prioritize counties for malaria prevention and control measures. Unfortunately, it is widely known that this type of data might have substantial noise when associated with small population sizes (as is the case for most Amazonian counties) and small case counts (as is the case for *Plasmodium falciparum*) [11]. The accurate mapping of *P. falciparum* is particularly critical from a public health perspective because, despite its relative rarity, *P. falciparum* infection tends to result in more severe outcomes than infections by *Plasmodium vivax*, the dominant type of malaria in this region [12]. Geospatial models have been widely used to ameliorate some of these problems, allowing for estimates of disease risk at a particular site to be influenced by neighbouring sites (i.e., borrowing of information across sites through spatial correlation).

In this article, the concept of borrowing information is taken one step further by allowing for estimates of *P. falciparum* disease risk to be influenced by *P. vivax* disease risk. Given that both Plasmodia are typically transmitted by the same set of vectors, sharing information across pathogens (i.e., modelling between-pathogen correlation) should improve predictions of *P. falciparum* disease risk. A novel multi-pathogen geospatial Bayesian model is introduced that accommodates for both between-site and between-pathogen correlation. This model aims to circumvent the problems associated with determining the API of counties with small population sizes and for relatively rare pathogens (e.g., malaria caused by *P. falciparum*). Model results uncover large-scale drivers of malaria incidence, spatially characterize malaria risk across the Brazilian Amazon and help identify key priority areas for malaria prevention and control interventions in the region.

## Methods

### Data

**Figure 1:**
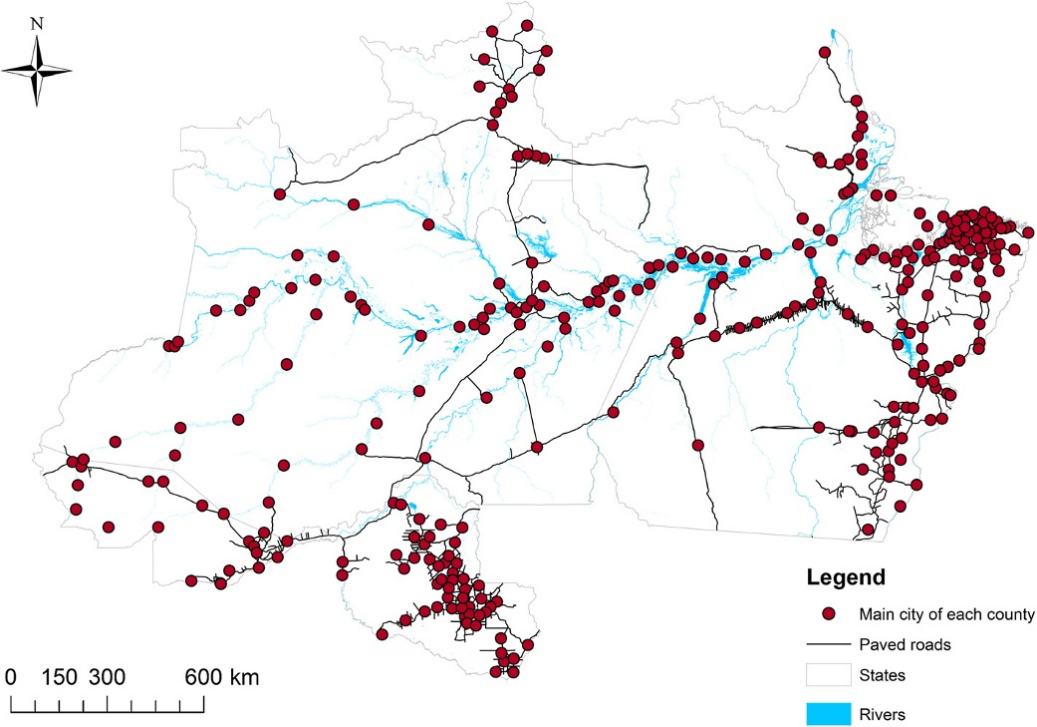
**Spatial distribution of the locations that originated the malaria data used in this article.**

The available malaria data consists of the number of malaria cases per month, county and pathogen (*P. vivax* and *P. falciparum*), collected from 2004 to 2008 by the Brazilian Government surveillance system in six Amazon states (i.e., Roraima, Rondonia, Para, Acre, Amazonas, and Amapa). These data, which encompass both autochthonous and allochthonous cases, diagnosed using microscopy, are available upon request from the Observatorio Clima e Saude [13]. In the absence of location information for the individual health facilities that gave rise to these malaria data, the spatial coordinates of each county’s main city were used to represent all malaria cases for that county. This assumption should be approximately valid given the large spatial-scale of the analysis and the fact that the majority of each county’s population is concentrated around these urban centres [14, 15] (see Additional file 1). Only 2% of counties had substantial missing data (>20 months with missing data out of a total of 60 months) and were excluded. In total, the study region encompassed 2.4 million malaria cases throughout an area of approximately 3.6 million sq km (Figure 1).

Several remotely sensed environmental covariates were gathered from multiple sources, including annual deforested and forested area, rainfall and length of dry season, and proximity to large water bodies. Annual deforested and forested area are based on a semi-automated annual analysis of Landsat imagery, provided by the Brazilian Institute of Space Research (INPE) [16]. Monthly rainfall data for 0.25^o^ × 0.25^o^ pixels come from the Tropical Rainfall Measuring Mission (TRMM) [17]. Precipitation data was used to calculate average monthly rainfall and the length of dry season (number of months with less than 100 mm of rainfall) [18, 19] for each year and county. To map large water bodies, a 250 m resolution global raster water mask, derived using MODIS and Shuttle Radar Topography Mission (SRTM) data was used [20]. In addition to these covariates, proximity to gold mining operations [21] and proximity to other large-scale infrastructure, such as roads and hydro-electric dams in operation, were also considered [22]. All covariates were assessed within a 50-km radius of each main city, encompassing the area where most of the population likely resides (see Additional file 1). Proximity-based covariates were transformed into dummy variables, taking on the value of 1 if the item (e.g., large water body, gold mining operation, road, or hydro-electric dam) was present within the 50-km buffer and zero otherwise.

Several socio-economic covariates based on the 2000 Brazilian Census, including income (i.e., average income of rural households), health (i.e., mortality rate for children under five years old), education (i.e., proportion of the rural population that is literate), and rural population size were incorporated in the model to understand the underlying large-scale drivers of malaria risk [23]. Finally, 2010 Census data were used to assess migration patterns, given by the proportion of people that arrived in the county over the previous three to five years. The population size for each county in each month was estimated by linearly interpolating the population counts from the 2000, 2007 and 2010 Brazilian Censuses. These population estimates were then used as offsets in the statistical model.

### Statistical model

A multi-pathogen geospatial model was created to infer malaria risk over the entire 3.6 million sq km Brazilian Amazon region, encompassing 303 counties. This model assumed that the number of malaria cases for county *i (*i = 1,…,303), month *t* (t = 1,…,60) and pathogen *j* (j = 1,2) came from an over-dispersed Poisson distribution:


where *N*_*it*_ is the estimated population. At a second stage, the log-malaria risk *w*_*itj*_ is given by:


where *α*_*iyj*_ is the mean annual log-malaria risk for location *i*, year *y* (y = 1,…,5), and pathogen *j*. Here *v*^2^ captures month-to-month within-year variation. Assuming a normal distribution for the second-stage model is standard for Bayesian generalized linear models [24]. At a third stage, *α*_*iyj*_ is given by:


where  is the design vector, containing covariate information, **β**_*j*_ is the vector of regression parameters for pathogen *j*, and *e*_*iyj*_ is the residual. Different assumptions can be made regarding the nature of these residuals. For instance, if one assumes that  and that these residuals are independent, the resulting model would be equivalent to two independent regressions, one for each pathogen *j*.

Similar to most geospatial models, the correlation of the residuals for sites *i* and *k* was assumed to be given by *Cor*[*e*_*iyj*_, *e*_*kyj*_] = exp(-*ϕD*_*ik*_), where *D*_*ik*_ is the distance between these sites. This expression describes the correlation among residuals within the same year *y* and pathogen *j* as a decay function of the distance between sites *i* and *k*. The innovation in the proposed model consists of further assuming that the correlation for pathogens *j* and *l* is given by *Cor*[*e*_*iyj*_, *e*_*iyl*_] = *ρ*. This assumption allows for information on pathogen *j* to influence, and potentially improve, inference on pathogen *l*. Such a feature might be useful if incidence of these diseases is likely to be correlated (e.g., because they are transmitted by the same vector).

These assumptions on the correlation structure of the residuals can be more succinctly described using a multivariate normal distribution, where the vector **α**_*y*_, which includes the mean annual log-malaria risk from year *y* for all counties and pathogens (i.e., *α*_*iyj*_ for all *i* and all *j*), is given by:


where ⊗ denotes the Kronecker product. The original design matrix **X**_*y*_ contains information regarding the covariates for all sites in year *y*, where each continuous covariate was standardized to have zero mean and standard deviation of one and enters the model with a linear and quadratic term. Based on the design matrix,  is defined to be equal to  and, correspondingly, the associated vector of slope parameters β* is defined to be equal to , where **β**_1_ and **β**_2_ are the vectors for pathogens 1 and 2, respectively, This specification for  and β* allows for the different pathogens to have different regression parameters. The covariance matrix of this multivariate normal, given by *σ*^2^**P**(*ρ*) ⊗ **S**(*ϕ*), is separable into an overall variance *σ*^2^ parameter, a pathogen correlation matrix **P**(*ρ*), and a spatial correlation matrix **S**(*ϕ*). Similar separable correlation matrices are often used for spatial-temporal models [24].

Priors for these parameters are given by


where **I** is the identity matrix. Priors on the variance parameters *v*, *σ* were chosen based on the recommendation from [25]. Finally, similar to [26], a discrete support for *ϕ* was assumed to aid mixing of a Monte Carlo Markov Chain (MCMC) algorithm. This was implemented by assuming a uniform prior over a set of values for φ, where these values were chosen based on a preliminary analysis.

To determine the large-scale drivers of malaria risk in the region, an adaptation of the methodology described in [27] was used to first determine which socio-economic and environmental covariates were significantly associated with malaria incidence. This helped avoid over-fitting the model given the large number of covariates being evaluated. The model described above was then fit using the predictors identified to be important by that analysis. Spatial predictions, on the other hand, required values for all covariates at all prediction locations. Thus, only spatially explicit environmental covariates were used for spatial predictions, ignoring the socio-economic covariates. Finally, a cross-validation exercise (described below) was performed to determine the out-of-sample predictive skill of the model when applied to create interpolated surfaces of *P. falciparum* disease risk. Again, only spatially explicit environmental covariates were used for this validation exercise. For both the spatial predictions and the cross-validation exercise, linear and quadratic terms of these spatially explicit environmental variables were included, whether or not they were significant.

### Cross-validation

The predictive ability of the proposed model was compared to that of alternative models typically employed to analyse this type of data. The first alternative model consists of independent regression models for each pathogen, assuming that sites are independent. This is equivalent to setting the parameters ρ and φ to zero and a very large positive number, respectively. The second alternative model adopts a traditional geospatial model that accounts for spatial correlation but still models pathogens independently. This is equivalent to setting the parameter ρ to zero. For completeness, a third alternative model was included, which assumes that sites are independent (i.e., φ is set to a very large positive number) but allows for correlation between pathogens (i.e., ρ is estimated). Table 1 describes the set of compared models, where model 4 ‘Pathogen + Spatial’ denotes the proposed model described in the section ‘Statistical model’.

**Table 1:** **Description of the models compared in the ten-fold cross-validation exercise**

Models	Assumptions	Parameters
1 – No correlation	No spatial and no pathogen correlation	*ρ* = *0*; *Φ* > > 0
2 – Spatial	Accounts for spatial correlation but not for pathogen correlation	*ρ* = *0*; *Φ* estimated
3 – Pathogen	Accounts for pathogen correlation but not for spatial correlation	*ρ* is estimated; *Φ* > > 0
4 – Pathogen + Spatial	Accounts for pathogen and spatial correlation	*ρ* and *Φ* are both estimated

To determine the out-of-sample predictive skill of these models for *P. falciparum*, a ten-fold cross-validation was performed. This procedure entails the random exclusion of 10% of *P. falciparum* observations and the use of the rest of the data to fit the four models described in Table 1. Then, left-out observations were predicted with the fitted models. This was repeated ten times, each time leaving out a distinct subset of the data. Predictive skill was assessed using two criteria: (1) the proportion of left-out observations that were within the 95% predictive credible interval (95% CI coverage). For good models, the proportion of left-out observations within this interval should be close to 95%; and, (2) mean-squared-error (MSE) of the predicted malaria incidence per person. This criterion takes into account both bias and uncertainty in predictions and smaller MSE values indicate better models. All models were fit using a customized Gibbs sampler, with Metropolis-within-Gibbs steps for non-conjugate conditional distributions. All analyses were conducted in R [28] and the R code to fit the statistical model is available upon request.

### Hotspot and coldspot definitions

To complement the geospatially modelled malaria risks maps, hotspots and coldspots were mapped as a way to take into account both mean disease risk and its associated uncertainty. Hotspots are defined as sites that were predicted to have significantly higher disease risk than average. In other words, site *i* was deemed to be a hotspot for pathogen *j* if the 95% credible interval of *α*_*iyj*_ did not straddle  (i.e., the average disease risk across all years and counties for pathogen *j*). Furthermore, persistent hotspots are those areas classified as a hotspot in all five years, intermittent hotspots are areas classified as a hotspot in at least one but not all years, and potential hotspots are areas predicted to have very high malaria risk (mean risk greater than 95% of the risk in all locations) but whose 95% CI encompassed the average risk. Similar definitions were applied for coldspots (i.e., areas with significantly lower disease risk).

## Results

### Cross-validation results

**Figure 2:**
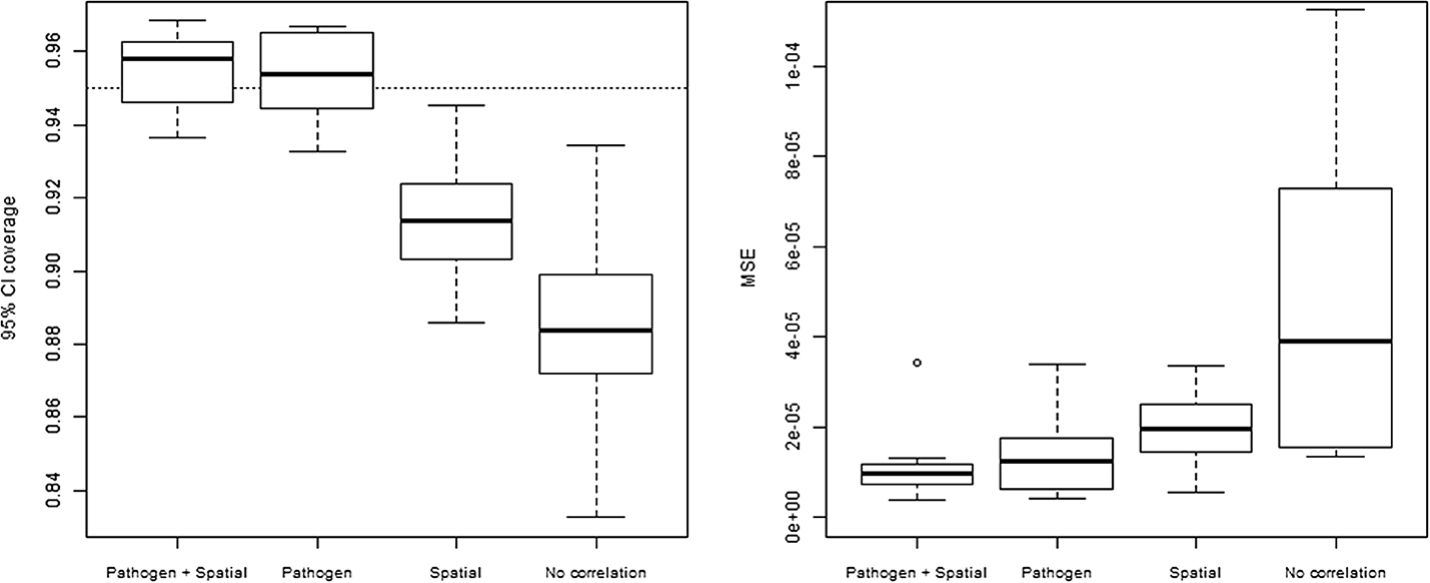
**The ‘Pathogen + Spatial’ model had the best out-of-sample predictive skill.** Left panel depicts 95% CI coverage, defined as the proportion of out-of-sample observations that fell within the estimated predictive 95% CI. Dotted horizontal line depicts the target CI coverage. Right panel depicts the Mean Squared Error (MSE) for each model. A description of each model is provided in Table 1.

Results clearly indicate that allowing for both spatial and between-pathogen correlation (‘Pathogen + Spatial’) yields the best out-of-sample predictive skill. The 95% CI from this model’s predictive distribution captured out-of-sample observations 95% of the time, similar to the model that only accounted for pathogen correlation (‘Pathogen’). On the other hand, the models that accounted only for spatial correlation (‘Spatial’) or no correlation at all (‘No correlation’) missed the out-of-sample observations more often (left panel in Figure 2). Better performance of the ‘Pathogen + Spatial’ model, based on the 95% CI coverage criterion, did not come at the expense of predictability. Rather, this model had the smallest MSE (a measure that combines bias and variance), again followed closely by the model with only pathogen correlation (‘Pathogen’) (right panel in Figure 2).

Validation results suggest that between-pathogen correlation is more important for out-of-sample predictive skill for this dataset than spatial correlation (i.e., compare the drop in out-of-sample predictive ability of the ‘Spatial’ model that neglects pathogen correlation relative to that of the ‘Pathogen’ model that neglects spatial correlation). These findings indicate that data on *P. vivax* incidence can substantially improve predictions for *P. falciparum* incidence.

### Malaria findings

**Figure 3:**
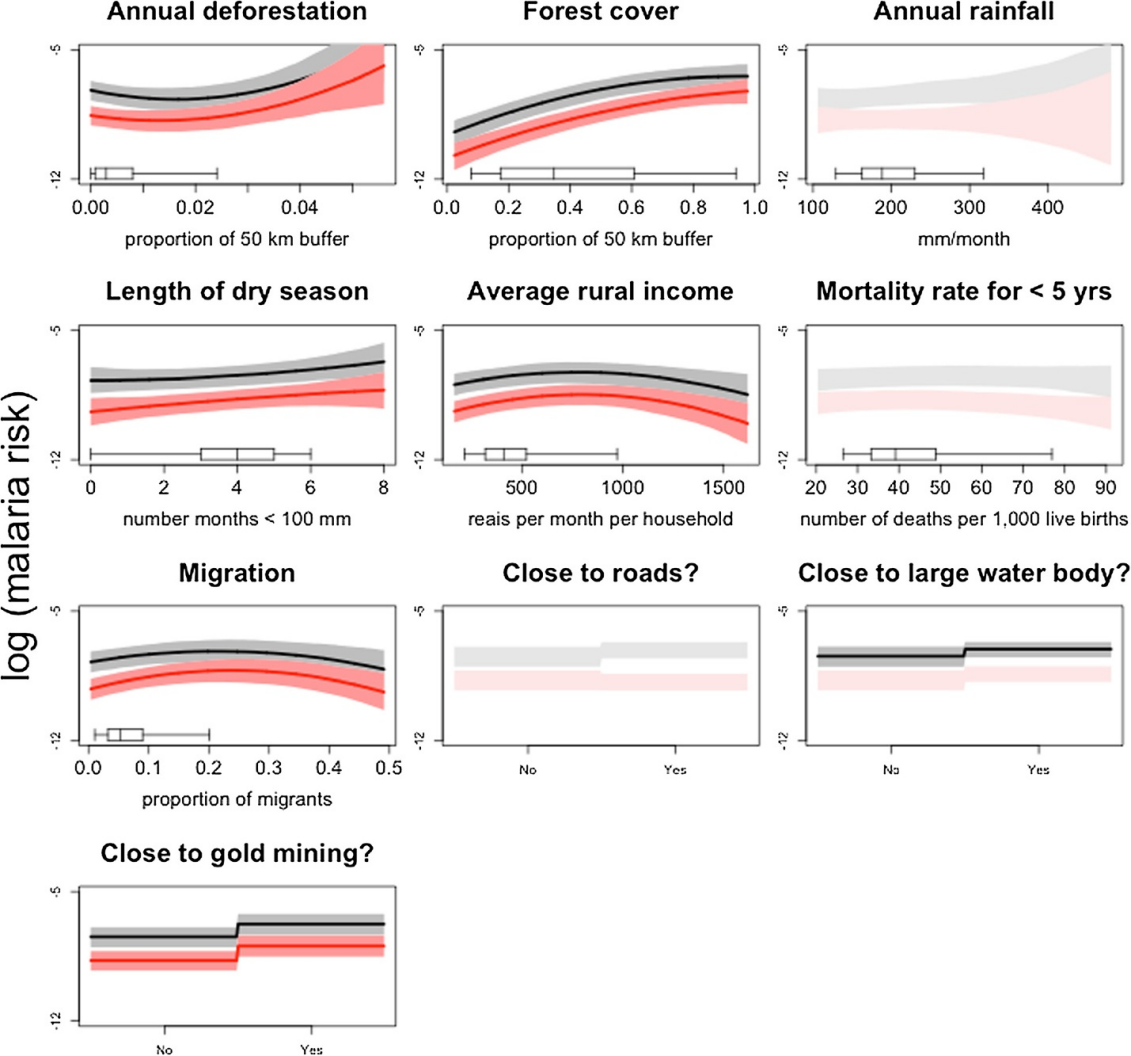
**Malaria risk factors for** ***Plasmodium vivax*** **(black) and** ***Plasmodium falciparum*** **(red).** Each panel depicts the estimated relationship between each covariate and log-malaria risk *α*_*iyj*_. Significant relationships are depicted with lines, which represent the median of the posterior distribution. Polygons are 95% CIs. Boxplots show the distribution of the corresponding covariate.

As in earlier work [6], the main factor driving malaria incidence was extent of forest cover (Figure 3), suggesting that increased human contact with forested areas (e.g., for fishing, hunting and timber logging) may enhance malaria risk. Another important predictor of malaria incidence was proximity to gold mining operations. Because high migration rates are often associated with artisanal gold mining operations, the positive association between migration and malaria risk found in the data provided further evidence of the role of gold mining activities in increasing malaria incidence throughout this region.

Average income of rural households and length of dry season were positively associated with malaria risk. Regions with higher average rural income may have greater access to health posts, potentially increasing malaria reporting rates relative to poorer counties. Findings regarding dry season length suggest that the formation of small and/or isolated water bodies when rivers recede during the dry season likely increases mosquito breeding habitat. Deforestation rate, annual rainfall, rural population size, rural literacy rate, child mortality rate (< five years), and proximity to roads or large water bodies, had relatively little effect on malaria risk. Surprisingly, no significant effect of proximity to hydro-electric dams was found, which could be due to the relatively coarse scale of the analysis or because of the enhanced malaria prevention and control programmes in these regions. Parameter estimates with associated 95% CIs for the final model are given in Table A1 in the Additional file 1. The model’s between-pathogen correlation parameter was high (E[ρ] = 0.86 with 95% CI 0.85-0.88) supporting the conclusion from the validation exercise that models accounting for between-pathogen correlation improve predictive performance. On the other hand, spatial correlation was small (< 0.16) for sites 100 km apart and negligible (< 0.05) for sites more than 160 km apart.

**Figure 4:**
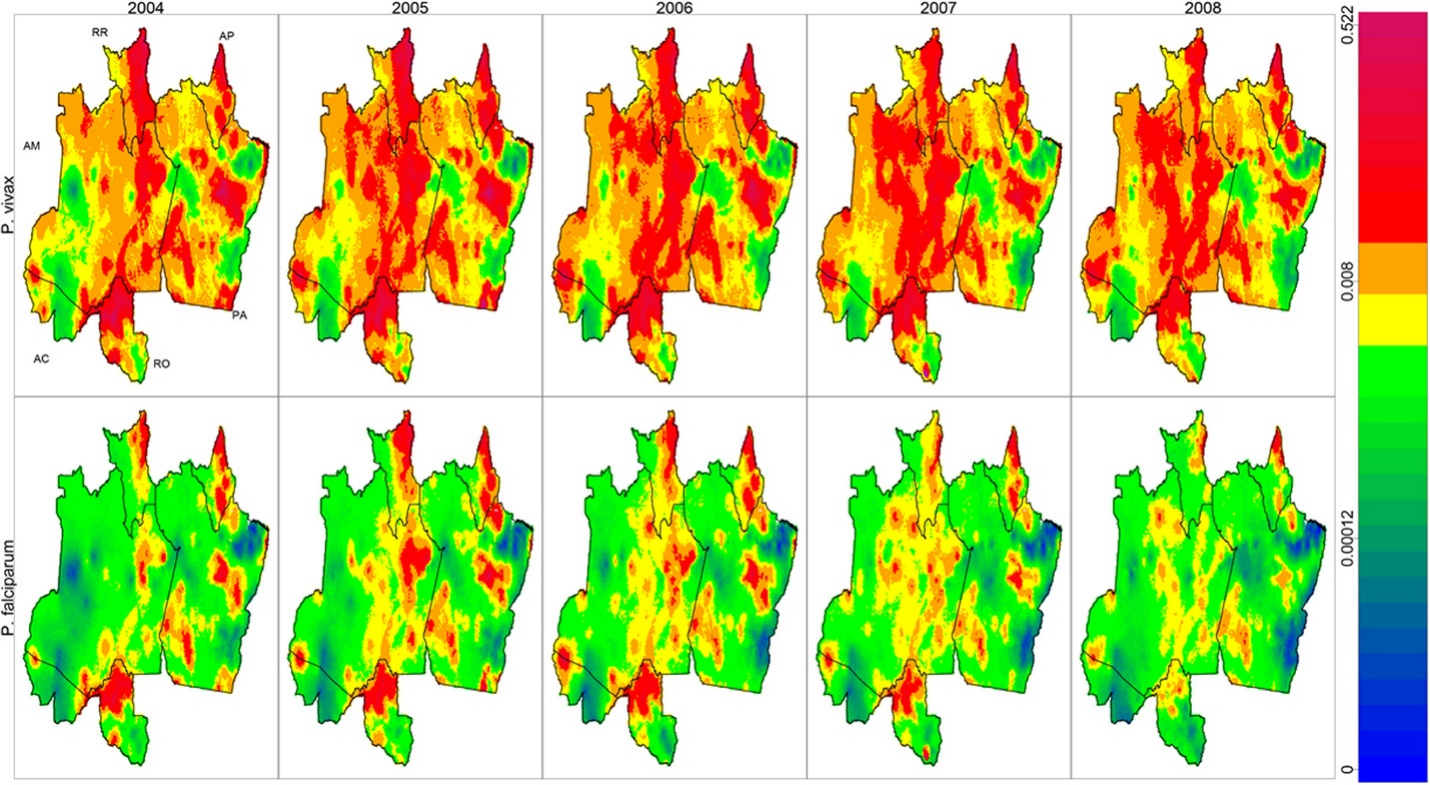
**Smooth surfaces of predicted malaria risk in the Brazilian Amazon.** Upper and lower panels show results for *P. vivax* and *P. falciparum*, respectively, while results for 2004 to 2008 are shown from left to right. Letters in the upper left panel refer to state names (AC = Acre, AM = Amazonas, RR = Roraima, AP = Amapa, PA = Para, and RO = Rondonia). The same colour scheme was used for all panels, where warmer colours indicate higher malaria risk. Numbers in colour key are predicted malaria incidence per capita per month.

**Figure 5:**
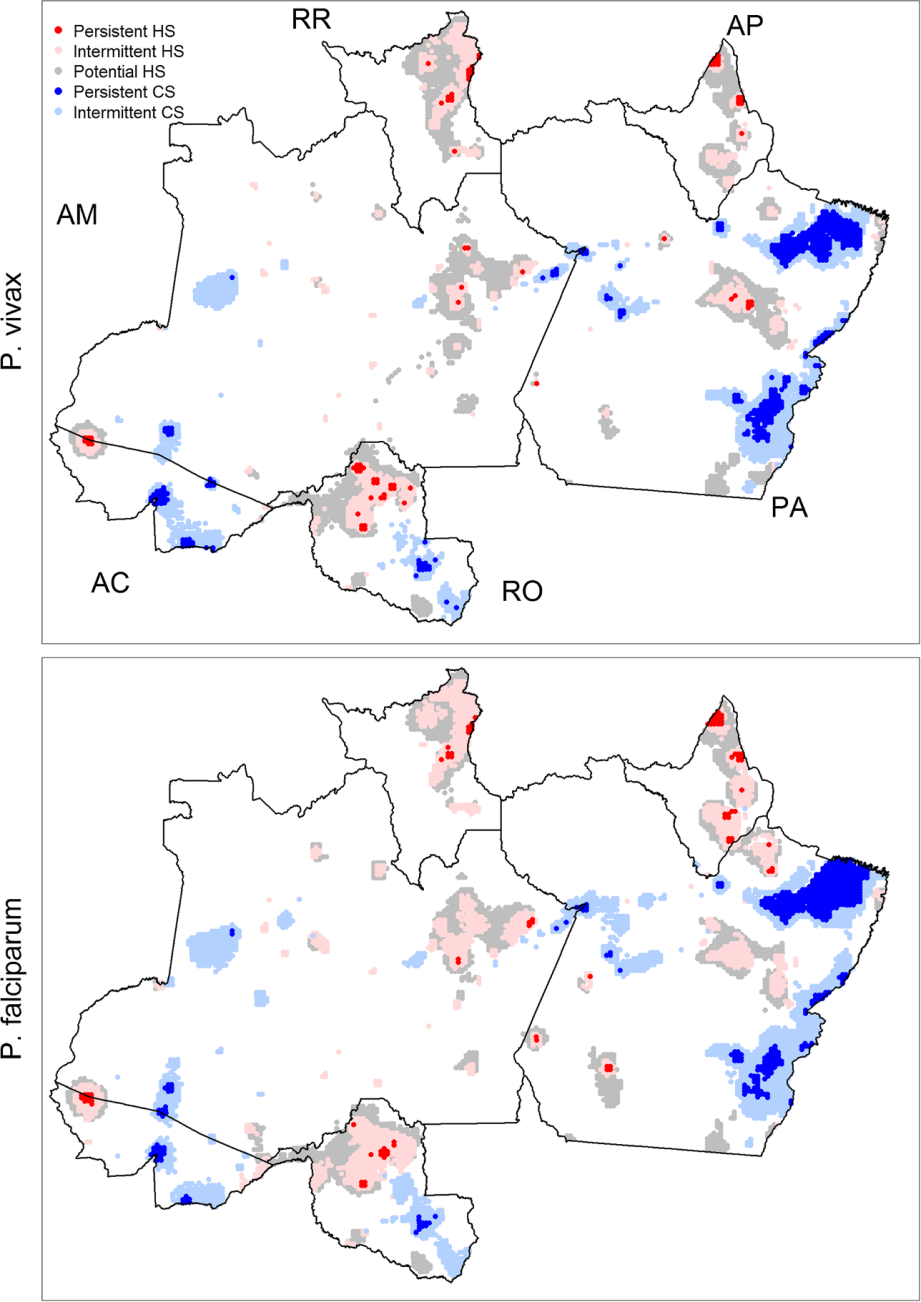
**Coldspots (CS) and hotspots (HS) reveal substantially different spatial patterns than Figure** 4 **.** Upper and lower panels show results for *P. vivax* and *P. falciparum*, respectively. Persistent hotspots (red) are areas that were classified as hotspots throughout the five-year study period (2004-2008). Intermittent hotspots (light red) are areas that were classified at least once as a hotspot but not in all five years. Analogous definitions were used for persistent (blue) and intermittent (light blue) coldspots. Finally, potential hotspots are shown in grey. Letters indicate state names (AC = Acre, AM = Amazonas, RR = Roraima, AP = Amapa, PA = Para, and RO = Rondonia).

Spatial predictions revealed substantial heterogeneity in malaria risk (Figure 4). As expected, *P. vivax* risk was much higher than that for *P. falciparum*, and high-risk areas for *P. vivax* and *P. falciparum* showed considerable overlap. The northern states of Amapa and Roraima displayed consistently high *P. vivax* risk, while the remaining states were more heterogeneous, with areas of both low and high *P. vivax* risk. For *P. falciparum*, higher risk areas were widely dispersed, concentrated in northern Rondonia, western Acre, eastern Amazonas, Amapa, Roraima, and central Para. Although it is tempting to draw conclusions directly from the spatial patterns depicted in Figure 4, accounting for uncertainty in these predictions is paramount. Predictions for areas with extreme covariate values, small population sizes, and/or far from data collection sites will likely display much larger uncertainty. Thus, to account for both mean risk and prediction uncertainty, hotspots and coldspots were delineated based on the definitions provided earlier. Figure 5 displays intermittent and persistent hotspots (HS, light red and red) or coldspots (CS, light blue and blue) for *P. vivax* (upper panel) and *P. falciparum* (lower panel). The approximate locations for areas classified as persistent hotspots for *P. vivax* and *P. falciparum* are given in Table 2. Notice that the spatial patterns displayed in Figure 5 are substantially different from those depicted in Figure 4. For instance, Figure 4 depicts a high *P. vivax* risk in southeastern and northwestern Amazonas state, but Figure 5 reveals that these spatial predictions cannot actually be deemed hotspots due to the large uncertainty associated with these areas.

**Table 2:** **Approximate location of persistent malaria hotspots by state**

State (acronym)	Both	***P. vivax***only	***P. falciparum***only
Acre (AC)	Rodrigues Alves, Mancio Lima, Cruzeiro do Sul		
Amapa (AP)	Calcoene, Oiapoque, Tartarugalzinho		Ferreira Gomes, Mazagao, Porto Grande, Santana
Amazonas (AM)	Careiro, Guajara, Itapiranga	Iranduba, Presidente Figueiredo, Silves	Sao Sebastiao do Uatuma, Urucara
Para (PA)	Jacareacanga	Pacaja	Anajas, Chaves, Itaituba, Novo Progresso
Rondonia (RO)	Porto Velho, Rio Crespo, Alto Paraiso, Campo Novo de Rondonia, Cujubim	Machadinho d’Oeste, Candeias do Jamari, Jamari	
Roraima (RR)	Bonfim, Canta, Mucajai	Amajari, Normandia, Rorainopolis	

**Figure 6:**
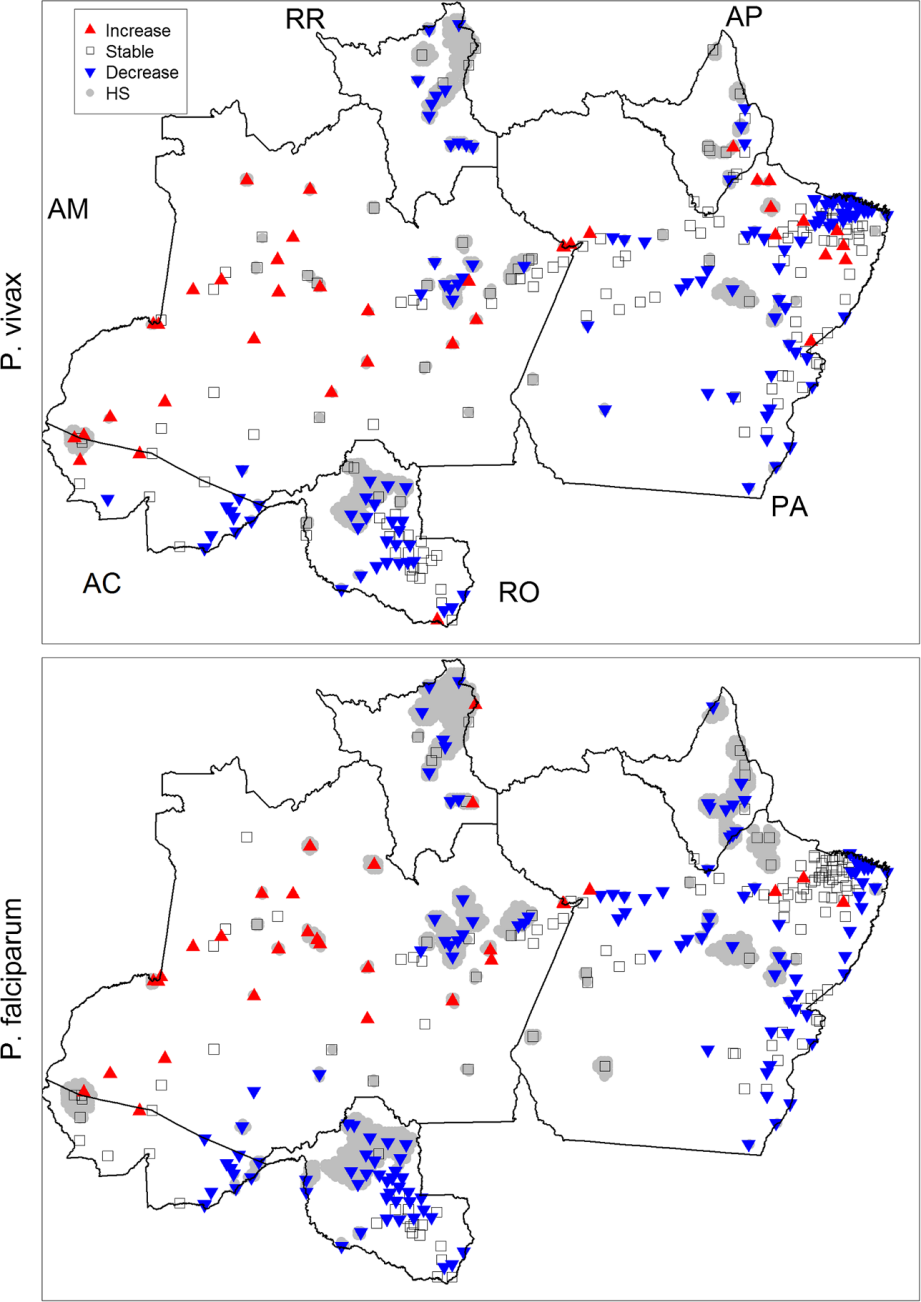
**Malaria risk is declining throughout most of the Brazilian Amazon region except for Amazonas state.** Areas where the estimated malaria risk significantly increased or declined from 2004 to 2008 are shown with red and blue triangles, respectively. Areas with no significant change (‘stable’) are shown with empty squares. Intermittent and persistent hotspots from Figure 5 are shown in grey in the background. Letters indicate state names (AC = Acre, AM = Amazonas, RR = Roraima, AP = Amapa, PA = Para, and RO = Rondonia).

Finally, changes through time were also examined by comparing estimates of log-malaria risk *α*_*iyj*_ for 2004 and 2008. This analysis revealed that mean malaria risk fell in 70-75% of the sites, with statistically significant declines being observed in 40-44% of the sites (Figure 6). Interestingly, sites with a significant increase of malaria risk are concentrated within the state of Amazonas, along with a handful of additional sites in northeastern Para and western Acre. Most of these sites are located in relatively remote areas, typically accessible only by boat, suggesting that logistical factors coupled with a strong influence of the riverine ecosystem may be hindering the prevention and control of malaria in these places. Since the comparison is only between 2004 and 2008, results should not be interpreted as a consistent trend (increase or decrease) in malaria risk over the study period. Finally, combining the hotspot analysis with these temporal results indicates that the most critical area for the Brazilian malaria prevention and control programme is in western Acre, including the counties of Rodrigues Alves, Mancio Lima, and Cruzeiro do Sul. These three counties consistently suffered above average malaria incidence while also exhibiting a significant increase in malaria incidence from 2004 to 2008.

## Discussion

Multiple large-scale infrastructure projects have been planned for future years to fully integrate the Amazon region (e.g., Planos de Crescimento Acelerado and Iniciativa para a Integracao de Infra-estrutura Regional da America do Sul – IIRSA). These projects include the expansion of the road network and the construction of multiple new hydro-electric dams, both in the Brazilian Amazon and in neighbouring countries [7, 8]. Interestingly, despite the widespread concern regarding increased malaria incidence due to the construction of dams (e.g., the Brazilian Government requires malaria studies prior to dam construction in the region), there was no association between proximity to these dams and malaria incidence. This lack of association might be due to the coarse scale of analysis and/or because of enhanced malaria surveillance and control in the vicinity of dams, typically initiated and sponsored by companies that build and operate these dams as a component of their impact mitigation plan. Not only did proximity to dams show no effect, forest cover results suggest that large-scale development projects likely induce a long-term decline of malaria incidence as a result of substantial deforestation associated with infrastructure development, similar to the conclusion in [6]. Nevertheless, these results should be viewed with caution due to the inherent limitations of observational studies and absence of long-term data. Furthermore, increased deforestation may have several other detrimental public-health effects. Finally, considering only the proximity to a dam reservoir or road fails to account for possible increases in malaria risk related to dam construction and/or road creation.

Proximity to gold mining operations exhibited a strong effect on malaria risk for both *P. vivax* and *P. falciparum*. Although this association has been described repeatedly in the literature e.g., [29–31], the detection of large-scale association between malaria incidence and gold mining is relatively rare [32]. It is important to note that the data consist of officially registered gold mining operations only. These data are used as a proxy for the location of gold mining activities in general because no comprehensive data on the location of small-scale artisanal gold mining operations (i.e., garimpos) at the scale of this analysis currently exists. Findings linking migration patterns with increased malaria incidence further support the importance of gold mining in increasing malaria risk, given that these activities typically spur considerable migration to the region.

Mapping disease risk is fundamental to public health, enabling strategic decision-making, such as spatially prioritizing resource allocation and deciding where to conduct more intensive interventions. Model predictions revealed substantial spatial heterogeneity across the Brazilian Amazon, highlighting regions that consistently have very high malaria risk. While these spatial predictions can be useful, relying on mean predictions alone to identify priority areas might be unreliable if their associated uncertainty is ignored. By identifying areas that are significantly and persistently above average, a handful of priority regions were highlighted (see Table 2). The importance of accounting for uncertainty in defining hotspots is particularly evident when comparing hotspots in Figure 5 with the traditional choropleth maps used by the Ministry of Health to identify areas of high malaria risk e.g., [33]. These discrepancies emphasize the critical role of uncertainty when creating interpolated surfaces of disease risk and the importance of carefully choosing the outcome to display in maps to effectively inform and guide public decision-making (see more discussion on this topic in [34]). The identified priority sites merit further scrutiny from the scientific community to better determine additional driving factors behind malaria risk in these persistent hotspots.

Results suggest that malaria incidence, both for *P. vivax* and *P. falciparum*, generally declined between 2004 and 2008 throughout most of the Brazilian Amazon region, except for Amazonas state. Sites with significant increases in the estimated malaria risk from 2004 to 2008 tended to be sites where transportation is typically limited to boats, suggesting that logistical factors may be hindering malaria prevention and control. Together with the hotspot analysis, this temporal analysis suggests that the Brazilian Government should prioritize the region in western Acre (i.e., Mancio Lima, Rodrigues Alves and Cruzeiro do Sul) for malaria prevention and control.

In this work, each county is represented by a single point at its urban centre (see Additional file 1). An alternative geospatial modelling approach that avoids the problem of determining the exact location of people and malaria infections relies on a conditionally autoregressive (CAR) prior for the spatial random effects. In such a model, spatial correlation is modelled based on an adjacency matrix, where two counties are deemed neighbours if they share a boundary. This approach is problematic in the Brazilian Amazon since human populations are highly clustered around county seats. Because counties are large, assuming that two counties are neighbours regardless of the distance between county seats seems unrealistic (e.g., [35]). Another problem with a CAR model is that the implied correlation matrix is often very counterintuitive [36]. Finally, CAR models do not allow for fine-scale geographical prediction, implicitly assuming that risk is the same throughout the county, and the resulting display of results using choropleth maps tends to over-emphasize larger counties. On the other hand, an important limitation of models like the one presented here is that implementation via Markov Chain Monte Carlo (MCMC) algorithms is computationally expensive due to the size of the covariance matrix [37]. While this problem is alleviated by using a separable correlation matrix, computational burden remains a critical issue, particularly if the model is expanded to include more than two pathogens and/or correlation between years. In future research, this problem might be tackled through the use of low-rank representations for spatial processes [38, 39].

An important limitation of the data used in this paper is that it lacks information on the total number of malaria examinations performed [40]. As a consequence, areas with a more comprehensive malaria surveillance network may apparently have higher malaria risk because of the detection of a greater number of cases. Thus, the high correlation between *P. falciparum* and *P. vivax* incidence identified by the model may be partly attributable to site-specific differences in surveillance intensity (i.e., enhanced surveillance will increase reported incidence of malaria cases from both pathogens). Regardless of the source of correlation between pathogens, joint modelling of data from multiple pathogens is still likely to be beneficial, particularly for diseases that are closely related (e.g., transmitted by the same vector, such as dengue, yellow fever and chikungunya). For instance, cross-validation results suggest that *P. vivax* incidence provides considerable information on *P. falciparum* incidence, potentially improving the ability to infer disease risk for this less common but more severe type of malaria. Only a few statistical models exploit information from data on multiple pathogens [41, 42], suggesting that the idea of integrating data from multiple pathogens to improve model predictions has been underexplored.

The gross underestimation of malaria symptomatic cases is not an issue in this study because malaria drugs are only available after obtaining positive microscopy detection in a government health facility. However, asymptomatic individuals and subpatent infections are likely to be missed by the data [43–45], and thus a map of infection risk might be substantially different from a disease risk map. Furthermore, *P. vivax* and *Plasmodium malariae* are commonly confounded in microscopic examination of thick blood smears [46–48], and as a result, findings regarding *P. vivax* are expected to be generally accurate but may falter in locations with high *P. malariae* incidence. Finally, in the absence of more systematically collected data on this topic, the role of concurrent local malaria interventions has been ignored.

## Conclusion

This article provides both methodological and epidemiological contributions. From a methodological perspective, the benefits of jointly modelling multiple pathogens for spatial predictions were illustrated, where abundant data from one pathogen can improve predictions of incidence of a related, rarer pathogen. Maps of mean disease risk (the usual end product of geospatial analyses) were contrasted with that of statistically significant disease clusters, highlighting the critical importance of uncertainty in determining disease hotspots. From an epidemiological perspective, large-scale drivers and spatial patterns of malaria incidence were revealed for the Brazilian Amazon. For instance, areas with higher forest cover and close to gold mining operations tended to have higher malaria incidence both for *P. vivax* and *P. falciparum*, suggesting that these are important large-scale drivers of disease risk in the region. Finally, key priority areas for malaria prevention and control were identified in this article. In particular, using both spatial and temporal results, the hotspot comprised of Mancio Lima, Rodrigues Alves, and Cruzeiro do Sul (Acre) should receive highest priority from the Brazilian national malaria prevention and control programme.

## Electronic supplementary material

Additional file 1: **Spatial clustering of people in the Brazilian Amazon region and parameter estimates for the final regression model.** (DOCX 26 KB)
