# Supplementary material for: Large-scale drivers of malaria and priority areas for prevention and control in the Brazilian Amazon region using a novel multi-pathogen geospatial model
Source: Malar J. 2014 Nov 20;13:443. doi: 10.1186/1475-2875-13-443 (PMC4247612; doi:10.1186/1475-2875-13-443)
Supplement: Supplementary file 1 — Additional file 1: Spatial clustering of people in the Brazilian Amazon region and parameter estimates for the final regression model. (DOCX 26 KB) [file 12936_2014_3600_MOESM1_ESM.docx]

**Additional File 1**

1. Spatial clustering of people around cities in the Brazilian Amazon region

Counties in the Amazon region can be huge (e.g., the Brazilian county of Altamira alone is larger in area than Greece). Nevertheless, the distribution of population is highly uneven, clustered around towns. This is clearly illustrated in the figure below, which uses the 2010 Brazilian census track information and assumes a 50 km buffer around each city. This figure shows the proportion of the population (y-axis) and the county area (x-axis) that fall within this 50 km buffer. If the population was evenly spread within each county, then the observations would fall roughly along the diagonal 1:1 line. However, for the vast majority of counties, such a buffer includes a much higher proportion of the population than its corresponding area would suggest (Fig. A1). Another way of seeing this spatial clustering around towns is to observe that 95% of the counties have >80% of its population living within this 50 km buffer. On the other hand, only 53% of the counties have >80% of its area within this buffer.

As a result of this uneven distribution of the population, people might live in a largely deforested area but a covariate that is assessed over the entire county might suggest that the area is heavily forested. For this reason, we strongly believe that assessing our environmental covariates within this 50 km buffer around each city is a better option than assessing these covariates over the entire county. Furthermore, we emphasize that a buffer of 50 km around towns includes a considerable fraction of populated rural areas. Thus, our results should not be interpreted as pertaining only to urban malaria.

**Fig. A1. Spatial clustering of the population around cities**. The proportion of the area (x-axis) and the proportion of the population (y-axis) for each county (circles) within a 50 km buffer around each city.


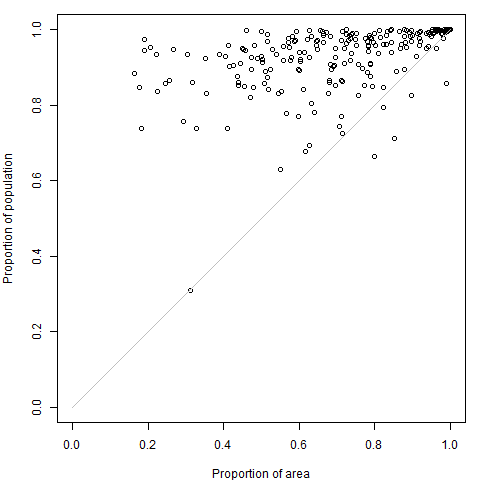


**Table A1. Parameter estimates for regression parameters in our final model.** Statistically significant parameters are emphasized in bold while non-significant parameters are depicted in grey. CI stands for credible interval.

|  | P. vivax | | | P. falciparum | | |
| --- | --- | --- | --- | --- | --- | --- |
|  | Median | 2.5% CI | 97.5% CI | Median | 2.5% CI | 97.5% CI |
| **Intercept** | **-7.45** | **-8.02** | **-6.94** | **-8.74** | **-9.28** | **-8.23** |
| **Annual deforestation rate (proportion of buffer)** | **-0.27** | **-0.49** | **-0.05** | -0.17 | -0.38 | 0.07 |
| **Annual deforestation squared** | **0.09** | **0.03** | **0.14** | **0.08** | **0.02** | **0.13** |
| **Annual forest cover (proportion of buffer)** | **1.01** | **0.83** | **1.19** | **1.09** | **0.91** | **1.27** |
| **Annual forest cover squared** | **-0.25** | **-0.39** | **-0.10** | **-0.18** | **-0.32** | **-0.03** |
| Annual mean precipitation (mm per month) | 0.15 | -0.07 | 0.40 | 0.02 | -0.23 | 0.27 |
| Annual mean precipitation squared | -0.01 | -0.10 | 0.08 | 0.00 | -0.10 | 0.09 |
| **Annual length of dry season (number of months with precipitation < 100 mm/month)** | **0.19** | **0.04** | **0.37** | **0.24** | **0.07** | **0.41** |
| Annual length of dry season squared | 0.04 | -0.04 | 0.12 | -0.02 | -0.10 | 0.07 |
| **Average income of rural households (reais per month)** | **0.22** | **0.08** | **0.38** | **0.32** | **0.16** | **0.46** |
| **Average income of rural households squared** | **-0.07** | **-0.11** | **-0.03** | **-0.09** | **-0.13** | **-0.05** |
| Mortality rate of children under 5 (per 1,000 live births) | 0.06 | -0.05 | 0.18 | 0.00 | -0.13 | 0.12 |
| Mortality rate of children under 5 squared | -0.04 | -0.10 | 0.02 | -0.05 | -0.11 | 0.01 |
| **Proportion of migrants** | **0.23** | **0.07** | **0.38** | **0.37** | **0.21** | **0.53** |
| **Proportion of migrants squared** | **-0.05** | **-0.07** | **-0.02** | **-0.07** | **-0.09** | **-0.04** |
| Main roads exist within 50 km (binary) | 0.34 | -0.02 | 0.72 | -0.10 | -0.47 | 0.28 |
| **Large water bodies exist within 50 km (binary)** | **0.39** | **0.03** | **0.76** | 0.34 | -0.02 | 0.70 |
| **Goal mining operations exist within 50 km (binary)** | **0.70** | **0.39** | **1.00** | **0.80** | **0.48** | **1.11** |
